# Arrowhead Composite Microneedle Patches with Anisotropic Surface Adhesion for Preventing Intrauterine Adhesions

**DOI:** 10.1002/advs.202104883

**Published:** 2022-02-20

**Authors:** Xiaoxuan Zhang, Guopu Chen, Yuetong Wang, Lu Fan, Yuanjin Zhao

**Affiliations:** ^1^ Department of Rheumatology and Immunology Nanjing Drum Tower Hospital School of Biological Science and Medical Engineering Southeast University Nanjing 210096 China; ^2^ Chemistry and Biomedicine Innovation Center Nanjing University Nanjing 210023 China; ^3^ Oujiang Laboratory (Zhejiang Lab for Regenerative Medicine Vision and Brain Health) Wenzhou Institute University of Chinese Academy of Sciences Wenzhou Zhejiang 325001 China; ^4^ Institute for Stem Cell and Regeneration Chinese Academy of Science Beijing 100101 China

**Keywords:** anisotropic surface, antiadhesion, biomaterials, microneedles, patches

## Abstract

Biomedical patches are considered as a promising strategy to help tissue repair and regeneration, prevent tissue adhesion, and reduce neighboring friction. Here, novel arrowhead composite microneedle patches (MNPs) are presented with anisotropic surface adhesion and growth factor encapsulation using a heterogeneous template replication approach for endometrium repair and intrauterine adhesions (IUAs) prevention. The arrowhead structures bring about interlocking between the microneedle (MN) tips and tissues, allowing these MNPs to steadily adhere to the tissues. Besides, benefitting from the cytoadhesive needle‐tip material and the antiadhesive base material, these MNPs possess anisotropic surface adhesion and can facilitate cell adhesion on one surface to repair damaged tissues while restrain tissue contact on the other to prevent adverse adhesion. In the meanwhile, the encapsulated growth factor can be delivered through the MNs to the deep tissue, further accelerating tissue repair. Additionally, as the bases are soft and their patterns are highly tunable, the MNPs can change their shapes flexibly to adjust to the irregular morphology of uteri. It is demonstrated that these MNPs show good performances in treating injured endometrium and preventing IUAs of a rat model, indicating their great potential in versatile postoperative adhesion prevention and other clinical applications.

## Graphical abstract

Arrowhead composite microneedle patches with anisotropic surface adhesion and growth factor encapsulation are presented. The arrowhead structures and tunable shapes help with tissue adjustment and adhesion, while the anisotropic surface adhesion enables both damaged tissue repair and adverse adhesion prevention. Practical values of these microneedle patches are demonstrated in rat endometrium repair and intrauterine adhesions prevention.

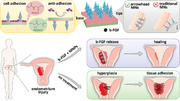

## 1 Introduction

Intrauterine adhesions (IUAs), a type of common gynecological diseases, show an uprising incidence with the increase of intrauterine surgeries and pose severe negative impacts on women's reproductive capacity and mental health.^[^ ^1^, ^2^ ^]^ To prevent and treat IUAs, a number of strategies have been developed, such as transcervical resection of adhesions,^[^ ^3^ ^]^ uterine cavity balloons,^[^ ^4^ ^]^ intrauterine scaffolds,^[^ ^5^, ^6^, ^7^, ^8^, ^9^, ^10^, ^11^ ^]^ and other methods.^[^ ^12^, ^13^, ^14^, ^15^, ^16^, ^17^ ^]^ Although with a certain therapeutic effect, these approaches are still faced with problems including high postoperative recurrence rates, easy dislocation, biological inertia, and so on. In contrast, biomedical patches have proven their superior antiadhesive abilities in medical fields,^[^ ^18^, ^19^, ^20^, ^21^, ^22^ ^]^ some of which have even been imparted with anisotropic structures to improve their performances.^[^ ^23^, ^24^, ^25^, ^26^ ^]^ However, most of the recent antiadhesive patches only possess simple function and have not realized the combination of antiadhesion and tissue repair. Besides, existing patches are not flexible enough to accommodate the special and complex geometry of the uterus. Thus, it is highly anticipated to provide biomedical antiadhesive patches with tissue conformal, non‐shifting, bioactive, and tissue‐repairing properties for IUAs prevention and treatment.

**Figure 1:**
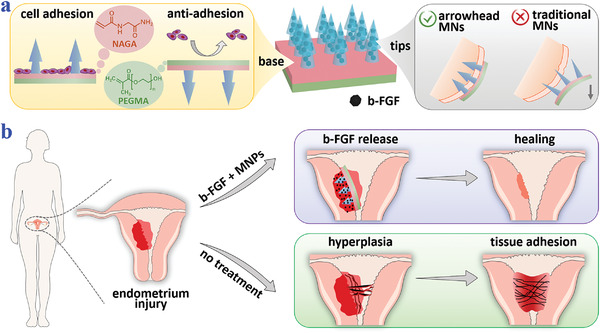
Schematic illustrations of the properties of the arrowhead composite MNPs and their applications in endometrium repair and IUAs prevention. a) The MNPs encapsulate b‐FGF in their tips and have bioactive NAGA and antiadhesive PEGMA as the base materials, which endow the MNPs with anisotropic surface adhesion. Besides, the arrowhead structures make the MNPs fix on the tissues. b) IUAs occur when the impaired endometrium receives no treatment, while the endometrium heals when treated with the MNPs.

Herein, we propose novel microneedle patches (MNPs) with arrowhead structures and anisotropic surface adhesion for the IUAs therapy, as schemed in **Figure** **1**. MNPs, which are featured by an array of microsized needle tips on a base, are playing an important part in biomedical patches.^[^ ^27^, ^28^, ^29^ ^]^ Such opportune sizes enable the MNPs to overcome the physiologic barriers and contact the deep tissue in a minimally invasive, painless, and harmless manner.^[^ ^30^, ^31^ ^]^ Due to their superior abilities in drug loading and delivery, MNPs can carry a variety of drugs (e.g., growth factors) to treat different diseases (e.g., tissue injuries).^[^ ^32^, ^33^, ^34^ ^]^ In addition, by introducing special physical structures to MNPs or designing chemical components of microneedle (MN) materials, satisfactory tissue fixation could be realized.^[^ ^35^, ^36^, ^37^, ^38^, ^39^ ^]^ However, majority of the current MNPs have not been exploited to the best advantage, with only their tip side exerting biomedical effects while the base side inoperative. Besides, it remains a challenge to apply the MNPs to the complicatedly shaped uterus.

In this paper, we present the desired growth‐factor‐loaded composite MNPs with arrowhead microstructures and anisotropic surface adhesion via a heterogeneous template replication method and demonstrate their values in repairing damaged endometrium as well as preventing post‐operation IUAs. By replicating differently sized templates in succession, the arrowhead structure could be fabricated, which made the tips interlock with tissues and thus adhere to the tissue without relative displacements. Besides, to impart the MNPs with anisotropic surface adhesion, growth‐factor‐containing methacrylated gelatin (gelMA) hydrogels, cytofriendly and bioactive *N*‐acryloyl glycinamide (NAGA) hydrogels, and antiadhesive poly(ethylene glycol) methacrylate (PEGMA) hydrogels were chosen as the tip material and the base materials, respectively. Attributed to this, the tip side of the resultant MNPs could promote cell adhesion and release drugs to accelerate tissue repairing, while the base side served as the antiadhesive surface to prevent adverse adhesion. Additionally, owing to the soft NAGA and PEGMA hydrogels and the tunable base designs, the MNPs could change their shapes flexibly and be patterned, thus adjusting to the irregular morphology of the uterus. It was demonstrated by a rat IUAs model that these MNPs performed well in impaired endometrium repair and IUAs prevention. All these features indicate the practical values of the composite MNPs in versatile postoperative adhesion prevention and other biomedical fields.

## 2 Results and Discussion

**Figure 2:**
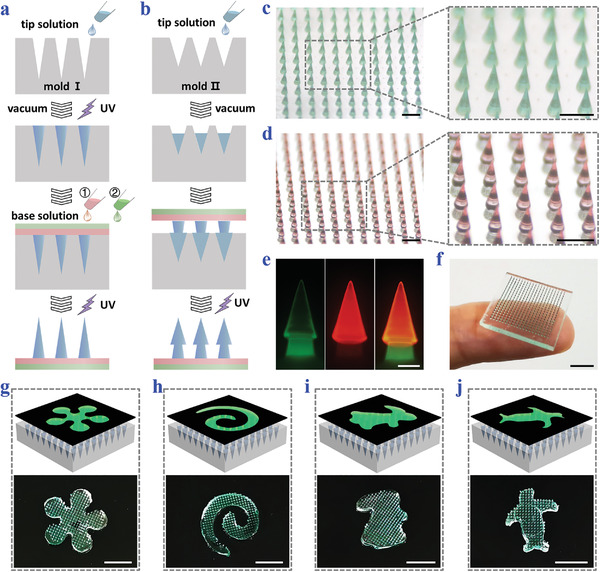
Fabrication and characterization of the arrowhead composite MNPs. a) Schematic illustrations of fabricating the straight‐head precursor MNP. b) Schematic illustrations of fabricating the arrowhead composite MNP on the basis of the precursor MNP. c) Optical images of the straight‐head precursor MNP. The tips are dyed green. d) Optical images of the arrowhead composite MNP. The second layers are dyed red. e) Fluorescence images showing the two layers of the arrowhead composite MNP. f) Photo of the arrowhead composite MNP placed on a finger. g‐j) Photos of arrowhead MNPs with g) flower, h) spiral, i) rabbit, and j) penguin patterns fabricated by using specially shaped photomasks. The tips of the MNPs are dyed green. Scale bars: 500 µm in (c, d), 200 µm in (e), and 0.5 cm in (f)–(j).

In a typical experiment, the arrowhead composite MNPs were fabricated by replicating heterogeneous templates stage by stage, as shown in **Figure** **2**. The first step was to prepare a straight‐head MNP as the precursor (Figure 2a). Specifically, gelMA prepolymer solution was added to the first negative mold (mold I) with spindly cavities to serve as the tip material. After vacuum treatment to fill the cavities, removal of the redundant tip solution, and UV exposure to solidify the tips, NAGA and PEGMA prepolymers as the base solutions were poured on the mold I one after another. By instantly connecting the base with tips via UV irradiation and detaching the mold I, the straight‐head precursor MNP would be generated (Figure 2c). Then such the precursor patch was inverted on a second negative mold (mold II) which had dumpy cavities with a small amount of tip solution in them (Figure 2b). They were solidified together under UV light and the arrowhead composite MNP was finally obtained (Figure 2d–f).

Significantly, these arrowhead composite MNPs were found to possess satisfactory uniformity, with heights centering around 760 µm, diameters concentrating at 230 µm, and interlayer spacing distributing between 200 and 236 µm (Figure S1, Supporting Information). Besides, the morphology of these composite MNPs could be facilely adjusted, so that they could well adapt to narrow uteri and various irregular wounded tissues, realizing customized and precise treatments. By employing a photomask to control the exposure area during UV irradiation, the MNPs could be molded into different sizes and shapes. For example, when the size of the photomask changed, MNPs with side lengths ranging from 0.5 to 1 cm could be easily fabricated, as displayed in Figure S2 in the Supporting Information. Besides, by altering the shape of the photomask, a variety of patterns including the flower, spiral, rabbit, and penguin could be introduced to the MNPs, as shown in Figure 2g–j.

**Figure 3:**
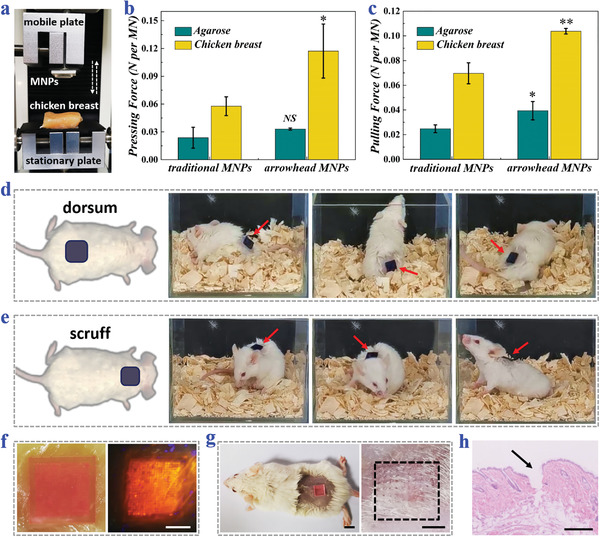
Tissue fixation and penetration of the arrowhead composite MNPs. a) Digital image of the mechanical test setup. b) Maximum forces required to press traditional straight‐head MNPs or arrowhead composite MNPs into agarose blocks or chicken breast (*n* = 3 for all groups; Student's *t*‐test was performed; the traditional MNPs were set as the baseline; *p < 0.05, NS: no significant). c) Maximum forces required to pull traditional MNPs or arrowhead MNPs out from agarose blocks or chicken breast (*n* = 3 for all groups; Student's *t*‐test was performed; the traditional MNPs were set as the baseline; *p < 0.05, **p < 0.01). d,e) Snapshots of moving mice with arrowhead MNPs applied to their d) dorsum skin and e) scruff skin. The MNPs are dyed blue for highlighting. f,g) Optical images of arrowhead MNPs penetrating f) the chicken breast or g) the mouse dorsal skin, and the corresponding microholes left after removing the MNPs. The MN tips are labeled red fluorescence. h) H&E staining of the mouse dorsal skin showing the MNP penetration. Scale bars: 0.5 cm in (f), 0.2 cm in (g), and 250 µm in (h).

Such arrowhead structures were expected to impart the composite MNPs with excellent tissue fixation abilities. To demonstrate this, the MNP was glued to the mobile plate of a mechanical testing instrument with its tips facing an agarose block or a piece of chicken breast, as shown in **Figure** **3**a. During the testing process, the MNP first moved downward until full penetration and then retreated until complete detachment. In the meanwhile, the pressing and the pulling forces applied to the MNP were recorded via a sensor. Results showed that compared to the traditional straight‐head MNP, it took more pressing forces for the arrowhead MNP to penetrate the agarose and chicken breast, which was caused by the uneven surfaces of the tips (Figure 3b). Whereas, the required maximum pressing forces of the arrowhead MNP were about 0.033 N per needle for agarose penetration and 0.117 N per needle for chicken breast insertion, respectively, and were easy to attain. Besides, based on the statistics of pulling forces, it was found to be much more difficult to pull the arrowhead MNP out from both the agarose and chicken breast than to pull the traditional MNP out (Figure 3c). Such enhanced fixation could be attributed to the interlocking between the arrowhead MNP tips and the agarose/chicken breast.

To show their tissue fixation capacities in real‐life scenarios, these arrowhead MNPs were applied to the dorsum and scruff of moving mice (Figure 3d,e). It was seen that the MNPs could well attach to the mouse whether it ran about, lifted its head, curled up, turned around, or stretched up, indicating the satisfactory tissue fixation. In addition, for characterizing the tissue penetration depth, these MNPs were dyed yellow and used to penetrate the translucent agarose (Figure S3, Supporting Information). Results showed that most of the needles could reach the depth of 600 µm and about one fifth could leave traces at 750 µm. Also, for displaying the penetration efficiency, fluorescence‐stained arrowhead MNPs were pressed on the chicken breast and mouse skin, and more than 90% of the needles were found to create microsized holes, as shown in Figure 3f,g. Besides, the MN‐penetrated mouse skin was further sampled and treated with hematoxylin‐eosin (H&E), which strongly demonstrated that the MNPs could well pierce the epidermis to reach the deep tissue (Figure 3h).

The tip material of the MNPs was determined to be gelMA hydrogel, which had sustainable drug release capacity. According to our previous experiments^[^ ^40^ ^]^ and other reported studies,^[^ ^41^ ^]^ gelMA was comparatively stable and took a relatively long time to degrade in vivo, avoiding the interference of excessive external factors on drug release. For the evaluation of their release profile of macromolecular protein drugs, fluorescein isothiocyanate‐labeled bovine serum albumin (FITC‐BSA), a model protein drug, was loaded in the tips of the MNPs. These MNPs were immersed in phosphate buffer saline (PBS) solution for simulating physiological environments and the amounts of released FITC‐BSA in PBS solution were measured at different time points. Judging from the release profile, over 60% of the total loading amounts were delivered at a rapid speed within the first 10 h (Figure S4, Supporting Information). Then the release gradually slowed down and reached its plateau. It should be mentioned that neither the release rate nor the cumulative release percentage had obvious relationship with the initial drug loading amount.

**Figure 4:**
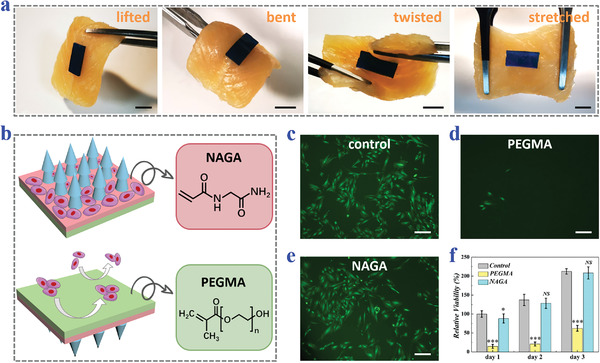
Tissue conformity and the anisotropic surface adhesion of the arrowhead composite MNPs. a) Photos of arrowhead MNPs sticking to the chicken breast when the chicken breast is lifted, bent, twisted, or stretched. The MNPs are dyed blue. b) Schematic illustrations showing the chemical components and cell adhesion of each side of the MNPs. c‐e) Fluorescence images of hE‐ADVs cultured on the c) plate wells, d) PEGMA hydrogels, and e) NAGA hydrogels. f) MTT results showing the relative viability of hE‐ADVs cultured on the plate wells, PEGMA hydrogels, and NAGA hydrogels on day 1, day 2, and day 3 (*n* = 8 for all groups; Student's *t*‐test was performed; the control group was set as the baseline; *p < 0.05, ***p < 0.001, NS: no significant). Scale bars: 0.5 cm in (a) and 200 µm in (c)–(e).

In addition to the tips, the base of the MNPs was also imparted with structural and biological functions. Benefitting from the soft, flexible NAGA‐PEGMA hydrogel base, the MNPs had the ability to conform to the attached tissue. For verification, the MNPs were applied to an ex vivo tissue. It was found that the MNPs could conformally adapt to the morphology change without falling off when the ex vivo tissue was lifted, bent, twisted, and stretched (**Figure** **4**a). Besides, since NAGA hydrogel mimicked the extracellular matrix as well as contained plenty of Gly motifs and hydrogen bonds, the NAGA‐composed MN upper base could contribute to cell adhesion and cell proliferation, thus promoting injury repair; on the contrary, due to the lack of cell adhesion sites on PEGMA hydrogel, the PEGMA lower base prevented cells from growing on its surface, which could avoid adverse tissue adhesion (Figure 4b). To prove such anisotropic surface adhesion of our MNPs, human endometrial adventitial cells (hE‐ADVs) were cultured on NAGA hydrogels, PEGMA hydrogels, and plate wells, respectively. The fluorescence images of live cells after 2 d clearly showed that hE‐ADVs could adhere to the NAGA hydrogel surfaces and grow as well as those on plate wells, while most of hE‐ADVs could not stay on the PEGMA hydrogel surfaces (Figure 4c–e). For further statistical analysis, 3‐(4,5‐dimethylthiazol‐2‐yl)‐2,5‐diphenyltetrazolium bromide (MTT) assay was carried out, which reflected cell viabilities on the three different surfaces. Such data were consistent with the fluorescence images, in which cells had similar viabilities on the NAGA hydrogel surfaces and plate wells, but showed the lowest viability value on the PEGMA surfaces (Figure 4f).

The biocompatibility of the presented MNPs should be investigated before they were applied in vivo. For this purpose, extracting liquid of gelMA or PEGMA/NAGA was employed to culture hE‐ADVs. It was found that the addition of gelMA extract or PEGMA/NAGA extract had no influence on the cell growth and proliferation, indicating the excellent cytocompatibility of the MNP materials, as shown in the fluorescence images and MTT results (Figure S5, Supporting Information). Additionally, the gelMA hydrogel and PEGMA/NAGA hydrogel were implanted under the skin of BALB/c mice to find out whether these materials would induce evident immune rejection and inflammatory reactions. After 2 weeks, the tissues around the implantation were sampled for histological analysis. Both H&E staining and Masson's trichrome staining showed clearly layered peripheral tissues, well‐maintained tissue structures, and almost no inflammatory cell infiltration (Figure S6, Supporting Information). All these results demonstrated that these biocompatible MNPs could safely function for in vivo applications.

**Figure 5:**
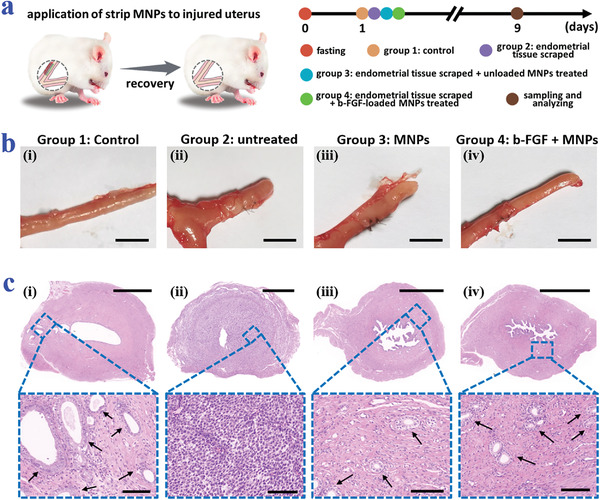
Performance of the b‐FGF‐loaded arrowhead composite MNPs in IUAs prevention in rats. a) Schematic illustrations and timeline showing the grouping and treatments on different groups. b) General views of the uteri of rats from the four groups. c) Corresponding H&E staining. The arrows point to the glands. Scale bars: 0.5 cm in (b), 1000 µm in the overall images of (c), and 100 µm in the magnified images of (c).

Based on all these features, the practical performances of these MNPs in tissue repair and adhesion prevention were further evaluated in a rat IUAs model. To establish such the model, the rats were anesthetized and their abdominal cavities were opened (Figure S7, Supporting Information). The uterus was then exposed, which was subsequently cut from the upper one‐third and scraped until it bled. The rats were randomly divided into four groups and the experiment protocol was illustrated in **Figure** **5**a. Specifically, the control group received sham operation and the injured uterus of the untreated group was sutured for self‐healing; while for the MNPs group and the fibroblast growth factor basic (b‐FGF) + MNPs group, MNPs without drugs and MNPs carrying b‐FGF were applied to the tissue lesions, respectively (Figure S7c,d, Supporting Information). Notably, to accommodate to the tubular structure of the uterus, the MNPs were fabricated into a strip shape. On day 9, the rats were sacrificed and the general views of their uteri preliminarily showed the successful establishment of the IUAs model using the above method as well as the evident recovery of the uterus under b‐FGF‐loaded MNPs treatment (Figure 5b).

**Figure 6:**
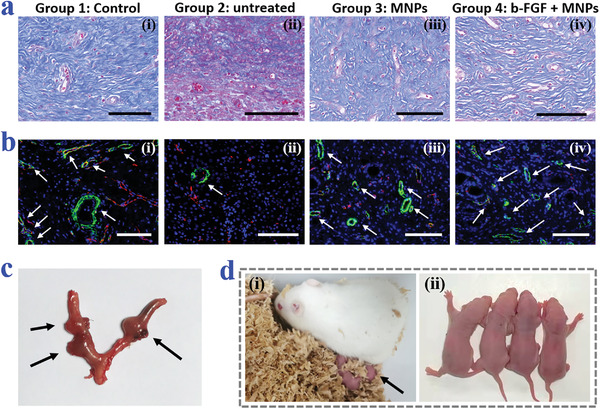
Histological staining analysis and reproductive outcomes. a) Masson's trichrome staining of the uteri of rats from the four groups. b) Corresponding immunofluorescence staining of CD31 and *α*‐SMA. The arrows point to the blood vessels. c) General view of the uterus containing embryos of the female rat in the b‐FGF + MNPs group. The arrows point to the rat embryos. d) Digital images of the female rat and its newborn babies. The arrow in (i) points to the baby rats. All scale bars: 100 µm.

To further show the adhesion degree and the repair of the endometrium, H&E staining was conducted on the collected uterus samples. H&E images together with the corresponding statistical analysis demonstrated that the b‐FGF + MNPs group had the best endometrium recovery and the largest number of glands compared to the untreated group and the MNPs group (Figure 5c and Figure S8a, Supporting Information). Also, both the MNPs group and the b‐FGF + MNPs group displayed the inhibition of the tissue adhesion, indicating the antiadhesion role of the MNPs. Besides, for revealing the collagen remodeling, Masson's trichrome staining was carried out. It was found that the collagen deposition of the b‐FGF + MNPs group was more regular and orderly than that of the untreated group and the MNPs group, showing better tissue regeneration (**Figure** **6**a). Additionally, immunofluorescence staining of CD31 and α‐smooth muscle actin (*α*‐SMA) was employed for detecting the neovascularization in the regenerative tissue. Obviously, the vascular density gradually decreased from the b‐FGF + MNPs group, the MNPs group, to the untreated group (Figure 6b). Consistent with the fluorescence images, the quantitative analysis of the vascular density clearly uncovered the angiogenesis ability of the b‐FGF‐loaded MNPs (Figure S8b, Supporting Information). Particularly, after 6‐week treatment, the female rats in the b‐FGF + MNPs group could successfully become pregnant and give birth to baby rats, demonstrating the actual values of these b‐FGF‐loaded MNPs in preventing IUAs and healing endometria (Figure 6c,d).

## 3 Conclusion

In summary, we have fabricated composite MNPs with the features of arrowhead microstructures and anisotropic surface adhesion, and encapsulated b‐FGF inside their tips for tissue repair as well as adhesion prevention. To generate these MNPs, a heterogeneous template replication strategy was employed, during which MNPs were replicated from differently sized templates in turns. The arrowhead structure allowed the MN tips to interlock with the tissues, made it difficult to detach them, and ensured steady tissue fixation. Besides, as the tips contained b‐FGF and the upper base was composed of bioactive, cytoadhesive NAGA, the needle‐tip side of the MNPs contributed to cell adhesion and could release drugs for injured tissue repair. Whereas, the lower base of the MNPs was made from antiadhesive PEGMA, which restrained cell adhesion on the reverse side and prevented adverse adhesion. In addition, due to the soft PEGMA/NAGA base and its changeable patterns which could be simply achieved using UV masks, the MNPs could well conform to the irregular morphology of different tissues. Based on these properties, these MNPs were demonstrated to be effective when applying to treat a IUAs rat model, indicating their practical values in impaired endometrium repair and IUAs prevention.

In addition to IUAs prevention, these composite MNPs are also promising in other postoperative adhesion prevention, like adhesions caused by abdominal and pelvic surgeries, and may provide treatment plans for various kinds of adhesions and injuries after improvements. In this case, the heights of the tips should be redecided to avoid secondary injury and the materials of the MNP base might be reconsidered dependent on the biodegradability. Besides, the MNPs presented at current stage only release loaded drugs in a passive and uncontrollable way. The capacity of controlled or on‐demand drug release is still waiting to be developed, which is bound to make the MNPs more intelligent and pragmatic. Additionally, during our animal experiment, the MNPs were applied invasively by opening the abdominal cavity and exposing the uterus. Thus, it is necessary to integrate MNPs with other medical apparatus or devices such as uterine balloons and uterus holders to achieve non‐invasive use for future clinical scenarios. For example, the MNPs might attach to the uterine balloons to be placed on the targeted sites. Also, the MNPs could be made into the shape of uterus holders to improve the conventional metal holders. Overall, faced with practical requirements in biomedical fields, this work presents a multidisciplinary approach by merging the design of microneedle and Janus structures in material science, the special properties of different hydrogels in chemistry, and the microfabrication techniques such as template replication in engineering. This study is anticipated to solve many problems in biomedical areas and arouse the development of many advanced devices. This topic will be of general importance to scientists and researchers from multiple backgrounds and inspire the combination of material, chemistry, engineering, and biomedicine.

## 4 Experimental Section

### Fabrication and Characterization of the Arrowhead Composite MNPs

In the first step, a straight‐head precursor MNP was fabricated. During this process, the aqueous solution of 25% w/v gelMA and 1% v/v 2‐hydroxy‐2‐methylpropiophenone (HMPP) was prepared as the MN tip solution, which was added to a negative mold with spindly cavities (about 800 µm in depth and 250 µm in width). After vacuum treatment for 5 min, the redundant tip solution was removed and the negative mold was exposed to UV light. The upper base solution (25% w/v NAGA prepolymer mixed with 1% v/v HMPP) was then poured on the above negative mold and the lower base solution (99% v/v PEGMA prepolymer with 1% v/v HMPP) was added subsequently. Both base solutions experienced UV irradiation together. By detaching the negative mold, the straight‐head precursor MNP was generated. In the next step, a small amount of tip solution was filled into another negative mold with dumpy cavities (about 550 µm in depth and 350 µm in width). Then the precursor MNP was inverted and inserted into the second negative mold. Finally, by solidifying them together under UV light and removing the negative mold, the arrowhead composite MNP was obtained. The optical images were taken by a stereomicroscope (JSZ6S, Jiangnan novel optics) equipped with a charge coupled device camera (Oplenic digital camera). The fluorescence images were captured using a biological microscope (FSX 100, Olympus). The digital photos were captured by a Huawei P30 pro mobile phone. During the preparation of MNPs for the follow‐up animal experiments, b‐FGF was loaded onto the MN tips by being blended in the MN tip solution at the concentration of 2 mg mL^−1^ for the purpose of accelerating endometrium repair.

### Fabrication of MNPs with Diverse Sizes and Shapes

To fabricate differently sized or shaped MNPs, a photomask was employed during each UV curing step. Taking the flower‐shaped arrowhead composite MNPs as the example, before exposing the tip‐solution‐containing negative mold I to UV light, a flower‐patterned photomask was used to cover the negative mold I, thus only allowing the UV light to pass through the flower sites and blocking other sites. After the base solutions were added, the same photomask was put on the same place and experienced UV irradiation. Additionally, the solidification of the tip solution in the negative mold II and the precursor MNPs was guided by the same photomask.

### Statistical Analysis of Penetration Depth and Efficiency

To clearly display the penetration depth, 5% w/v agarose blocks were made and the MNPs were stained with yellow water‐soluble pigments. The MNPs were pressed to the agarose with a finger. Based on the cross section images of the agarose, the penetration depths, which equaled to the depths of the created microsized holes, were measured by the software ImageJ. To calculate the penetration efficiency, the freshly bought chicken breast was cut to blocks, the mouse dorsal skin was prepared by anesthetizing the mouse, shaving the hair, and cleaning the bare skin with 75% ethanol, and the tips of MNPs were dyed by Rhodamine B. The MNPs were applied to the chicken breast and the mouse skins by finger, respectively. After removing the MNPs, the left microsized holes were recorded. By dividing the number of microsized holes in the chicken breast or mouse skin by the number of MN tips, the penetration efficiency was obtained. To demonstrate that the MNPs could overcome the epidermis barriers, the MN‐penetrated skin tissues were further sampled, sliced, treated with H&E staining, dehydrated, mounted in neutral balsam, examined by microscopy (BX51, Olympus), and photographed.

### Evaluation on the Anisotropic Surface Adhesion of the MNPs

25% w/v NAGA prepolymer (with 1% v/v HMPP) and 99% v/v PEGMA prepolymer (with 1% v/v HMPP) were added to different wells of a 24‐well plate and were directly solidified under UV light. After sterilization, hE‐ADVs were seeded on the NAGA hydrogel surfaces (NAGA group), the PEGMA surfaces (PEGMA group), and the plate wells (control group), respectively. These cells were stained by calcein acetoxymethyl ester (calcein‐AM) after 2 d. Besides, MTT assays were conducted on day 1, day 2, and day 3, and each group had eight parallels. Specifically, for each day and each well, the original culture medium was replaced by 1 mL new medium containing 10% v/v MTT solution. The cells were then incubated for 4 h and the medium was substituted by 500 µL dimethylsulfoxide (DMSO) for dissolving the generated formazan crystals. Finally, 100 µL of the DMSO solution was added to a 96‐well plate, and the optical density (OD) value at 490 nm was read out via a microplate reader (SYNERGY|HTX). The relative cell viability was obtained by comparing the OD values.

### Establishment, Treatment, and Effect Assessment of the Rat IUAs Model

The female Sprague Dawley (SD) rats with the same estrous cycle were randomly divided into four groups: the control group that received sham operation, the untreated group with the endometrial tissue scraped, the MNPs group with the endometrial tissue scraped and unloaded MNPs applied, and the b‐FGF + MNPs group with the endometrial tissue scraped and drug‐loaded MNPs applied. The rats were first anesthetized by intraperitoneal injection of 10% w/v chloral hydrate at the dose of 350 mg kg^−1^ body weight. After shaving the abdomen and disinfecting it with medical alcohol, the abdominal cavity was opened and the uterus was exposed. For the control group, the uterus was exposed for 10 min before suturing the abdomen with 4–0 suture. For other groups, the “Y”‐type uterus was picked out and was cut about 1.5 cm from the upper one‐third. Then the endometrium was scraped with a uterine scraper until it bled. For the untreated group, a 6–0 suture was used for suturing the uterus without other treatments. For the MNPs group and b‐FGF + MNPs group, stripe‐shaped MNPs carrying no drugs and carrying b‐FGF were placed in the injured endometrial tissues, respectively. On day 9, the rats were sacrificed, and their uteri were sampled and transversally sliced for the following H&E staining, Masson's trichrome staining, and immunofluorescence staining of CD31 and *α*‐SMA.

## Conflict of Interest

The authors declare no conflict of interest.

## Supporting information

Supporting Information

## Data Availability

The data that support the findings of this study are available from the corresponding author upon reasonable request.
