# Supplementary material for: Arrowhead Composite Microneedle Patches with Anisotropic Surface Adhesion for Preventing Intrauterine Adhesions
Source: Adv Sci (Weinh). 2022 Feb 20;9(12):2104883. doi: 10.1002/advs.202104883 (PMC9036003; doi:10.1002/advs.202104883)
Supplement: Supplementary file 1 — Supporting Information [file ADVS-9-2104883-s001.pdf]

Supporting Information

Arrowhead composite microneedle patches with anisotropic surface adhesion for preventing intrauterine adhesions

*Xiaoxuan Zhang, Guopu Chen, Yuetong Wang, Lu Fan, and Yuanjin Zhao**

1. Supporting Information text

Materials, cell lines, and animals: Poly(ethylene glycol) methacrylate (PEGMA) and 2-hydroxy-2-methylpropiophenone (HMPP) were purchased from Sigma-Aldrich (USA). Methacrylated gelatin (gelMA) was bought from EFL Co., Ltd (Suzhou, China). N-acryloyl glycinamide (NAGA) was from Macklin (Shanghai, China). Recombinant Human Fibroblast Growth Factor basic (b-FGF) was provided by Wenzhou Medical University. Fluorescein isothiocyanate-labelled bovine serum albumin (FITC-BSA) was obtained from Bersee Co., Ltd (Beijing, China). Agarose was bought from Aladdin (China). Phosphate buffer saline (PBS) was purchased from Servicebio Co., Ltd (Wuhan, China). All reagents were of analytical grade and used as received. Human endometrial adventitial cells (hE-ADVs) were given by the Drum Tower Hospital. Cells were cultured in Dulbecco's Modified Eagle Media: Nutrient Mixture F-12 (DMEM/F_{12}, from Gibco) mixed with 10% (v/v) Fetal Bovine Serum (FBS, from Gibco) and 1% (v/v) penicillin-streptomycin double antibiotics (Gibco) in an incubator (37 °C, 5% CO_2). Male BALB/c mice (6-8 weeks) and female Sprague Dawley (SD) rats (8-10 weeks) were provided by the Drum Tower Hospital. Animals were treated in strict accordance with the Beijing Administration Rule of Animals in China and have received approval from Animal Investigation Ethics Committee of the Drum Tower Hospital.

Mechanical tests of the MNPs: The pressing force and the pulling force were measured utilizing a mechanical testing instrument (ZHIQU Co. Ltd., Guangzhou, China). The MNP (with traditional straight-head tips or with arrowhead tips) was glued to the mobile plate of the instrument with its tips facing an agarose block or a piece of chicken breast, which was placed on the still plate. The moving speed of the mobile plate was about 0.1 mm/s. In the beginning, the MNP gradually moved down along with the mobile plate until the full penetration, during which time the pressing forces were recorded by a bundled sensor. The mobile plate then retreated to pull the MNP out and did not stop until complete detachment. At the same time,

the pulling forces were also recorded by the sensor. Using this method, the maximum pressing force and pulling force were acquired and compared.

Release profiles of FITC-BSA from the MN tips: FITC-BSA was mixed with the tip solution and the final concentrations of FITC-BSA were 0.5 mg/mL, 1 mg/mL, and 2 mg/mL, respectively. Then these tip solutions were made into MNP tips and immersed in PBS buffer solution at room temperature. At different time points, 100 μL of the PBS solution was drawn using a pipette to a 96-well plate (each had 5 parallels) and the same amount of new PBS solution was supplemented. The fluorescence intensities of the wells were read by a multimode plate reader with the excitation wavelength at 495 nm and the emission wavelength at 550 nm. Combining the real-time fluorescence intensity change and the concentration-intensity profile of FITC-BSA in PBS solutions, the cumulative release percentages could be plotted.

Investigation on the cyto-compatibility of the MNP materials: Extracting liquids of gelMA hydrogels (tip materials) and PEGMA/NAGA hydrogels (base materials) were first prepared by immersing the equally-sized hydrogels in 1 mL culture medium, respectively, and incubating them at 37 °C for 24 h. In the meanwhile, hE-ADVs were cultured in a 24-well plate for cell attachment. 24 h later, the culture medium was discarded and the cells were randomly divided to three groups (each had 8 parallels). Then the 24 h-aged culture medium was added to the control group, the 24 h-aged gelMA extracting liquid was added to the tip-material group, and the 24 h-aged PEGMA/NAGA extracting liquid was added to the base-material group. The cells were dyed with calcein-AM on day 2 and MTT assays were carried out on day 1, day 2, and day 3.

Subcutaneous material implantation tests: The BALB/c mice were anesthetized by inhaling isoflurane. By creating an incision in the mouse dorsalis, gelMA hydrogels or PEGMA/NAGA hydrogels with the size of 0.5 cm × 0.5 cm could be implanted under the skin. After 2-week implantation, the mice were sacrificed and the tissues around the implantation were sampled, fixed, dehydrated, embedded, sliced, and treated with both H&E staining and Masson's trichrome staining.

Statistical analysis: The control group was used as the baseline for normalization of the data. The data was presented as mean ± SD. The sample size (n) for each statistical analysis was mentioned in figure legends. Student's t-test was used to assess significant differences. $p < 0.05$ indicated that the difference was statistically significant. Software (SPSS 20.0, Origin 85, and Excel 2016) was used for statistical analysis.

2. Supporting Information figures

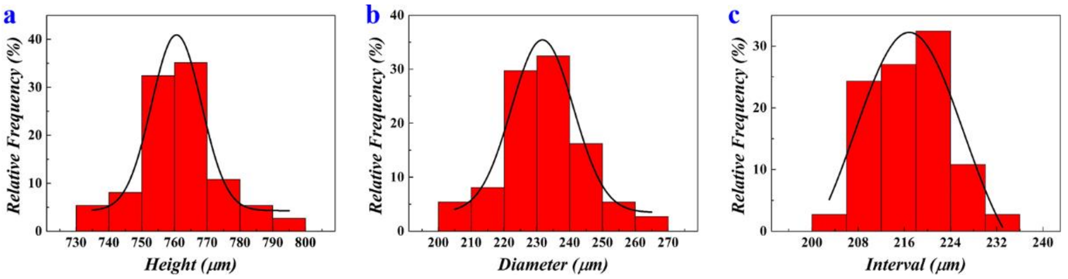

Figure S1. Uniformity of the arrowhead composite MNPs. (**a**) Height distribution of the MN tips (n=148). (**b**) Basal diameter distribution of the MN tips (n=148). (**c**) Layer-to-layer interval distribution of the MN tips (n=148).

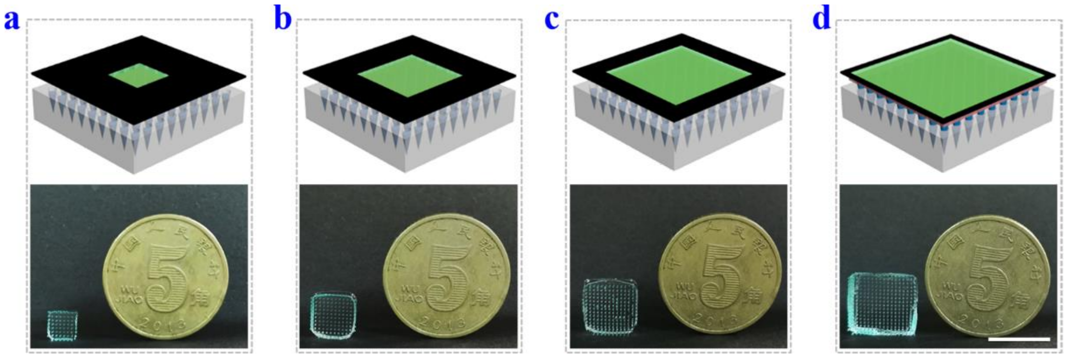

Figure S2. Square MNPs with various side lengths fabricated using photo masks with different sizes. Scale bar: 1 cm.

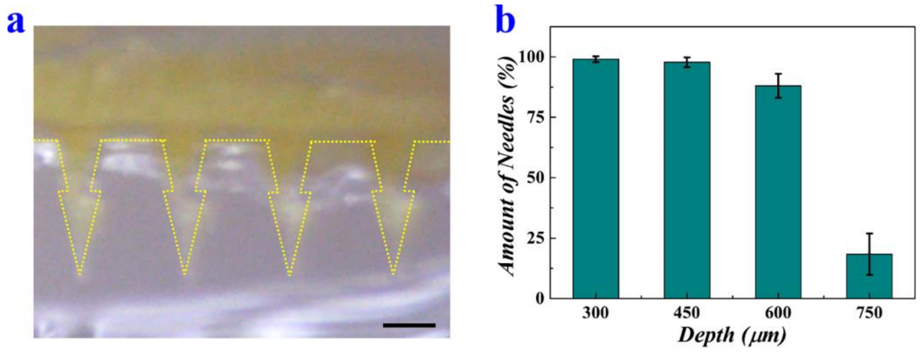

Figure S3. Penetration of the arrowhead composite MNPs in agarose. (**a**) Optical image of the tips of MNPs inside agarose. (**b**) Statistical analysis of the amount of MN traces at different depths of the agarose block (n=2400). Scale bar: 250 μm.

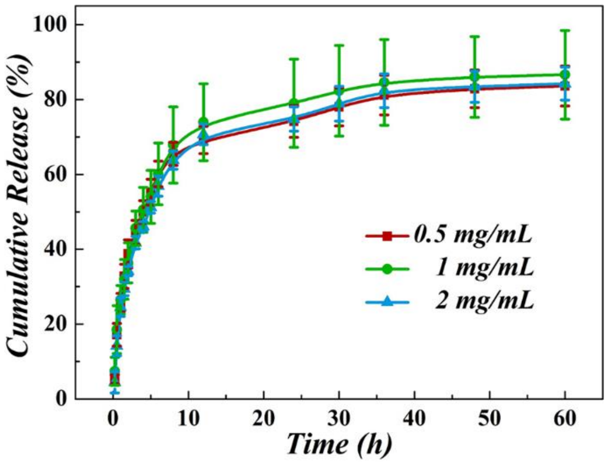

Figure S4. Cumulative release of FITC-BSA from the MN tips at different time points (n=5 for all groups). The initial FITC-BSA loading amounts are 0.5 mg/mL, 1 mg/mL, and 2 mg/mL, respectively.

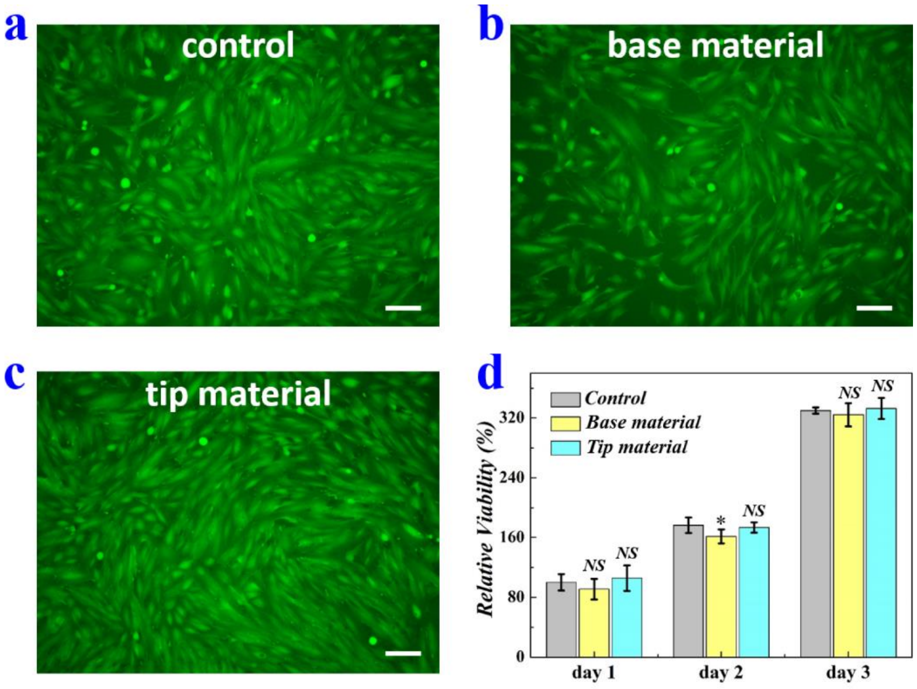

Figure S5. Cyto-compatibility assessment of the MNP materials. (**a-c**) Fluorescence images of hE-ADVs cultured in the ordinary culture medium (**a**), the base material extract (**b**), and the tip material extract (**c**), respectively. The live cells are dyed with calcein-AM. (**d**) MTT results showing the relative cell viability of the above three groups on day 1, day 2, and day 3 (n=8 for all groups; Student's t-test was performed; the control group was set as the baseline; * $p < 0.05$, NS: no significant). All scale bars: 100 μm.

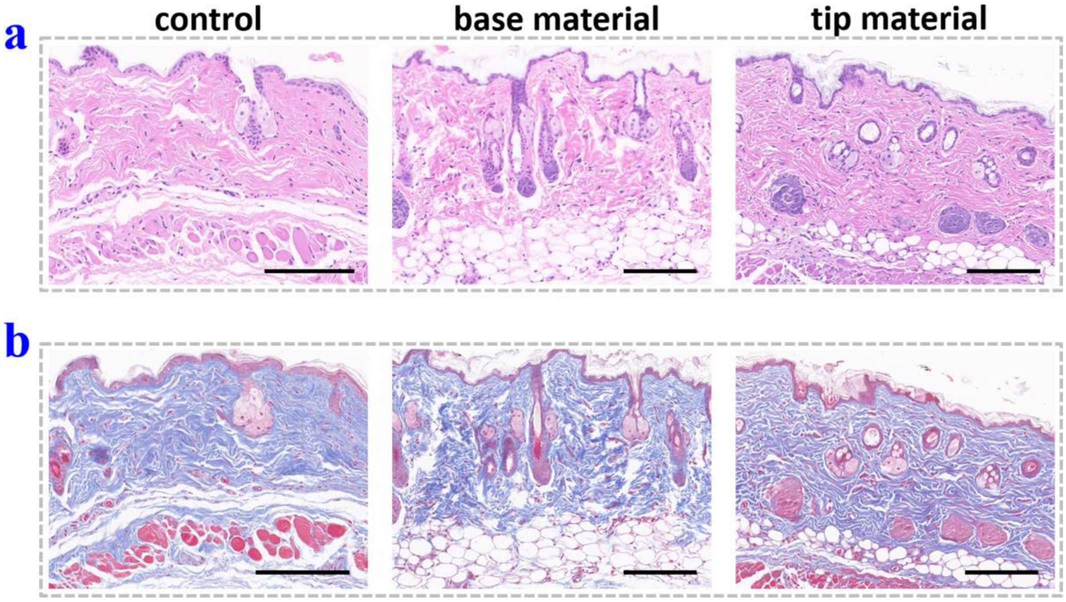

Figure S6. Histological analysis of the mouse skins around the implantation materials. (**a**) H&E staining of the mouse skins without material implantation (control), with base material implantation, and with tip material implantation, respectively. (**b**) Corresponding Masson's trichrome staining of the mouse skins. All scale bars: 150 μm.

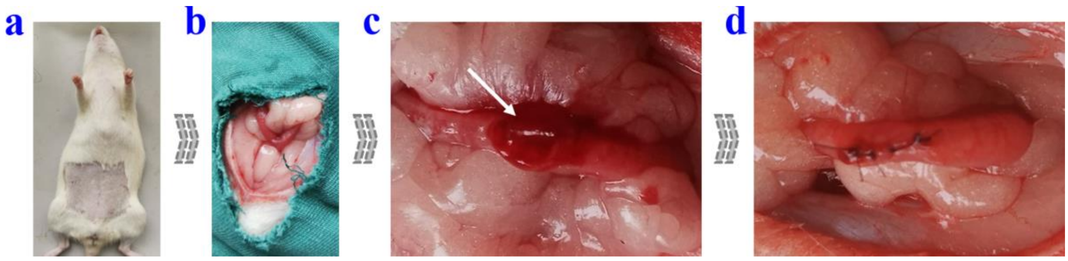

Figure S7. Digital images of the surgical procedure. (**a**) Anesthesia and skin preparation of the rats. (**b**) Uterus exposure. (**c**) Modeling and stripe-shaped MNPs implantation. The white arrow points to the MNPs. (**d**) Uterus suture.

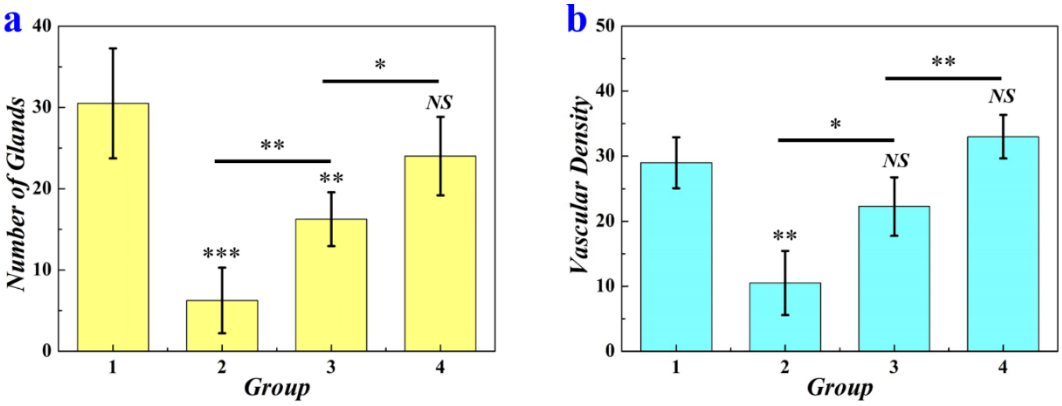

Figure S8. Statistical analysis of the rat modeling and treatment results. (**a**) The number of glands of rats from different groups (n=4 for all groups; Student's t-test was performed; Group 1 was set as the baseline; * $p < 0.05$, ** $p < 0.01$, *** $p < 0.001$, NS: no significant). (**b**) Vascular density of rats from different groups (n=4 for all groups; Student's t-test was performed; Group 1 was set as the baseline; * $p < 0.05$, ** $p < 0.01$, NS: no significant).

3. Author contribution

Y.J.Z. devised the idea and revised the Introduction Section. X.X.Z. designed the experiments, wrote the paper, and conducted the material experiments. G.P.C. was in charge of the animal experiments and checked the paper. Y.T.W. revised the manuscript and participated in scientific discussions. L.F. revised the manuscript and modified the grammar.
